# Impact of ten-valent pneumococcal conjugate vaccine on pneumonia in Finnish children in a nation-wide population-based study

**DOI:** 10.1371/journal.pone.0172690

**Published:** 2017-03-01

**Authors:** Arto A. Palmu, Hanna Rinta-Kokko, Hanna Nohynek, J. Pekka Nuorti, Terhi M. Kilpi, Jukka Jokinen

**Affiliations:** 1 Department of Public Health Solutions, National Institute for Health and Welfare, Tampere, Finland; 2 Department of Public Health Solutions, National Institute for Health and Welfare, Helsinki, Finland; 3 Department of Health Security, National Institute for Health and Welfare, Helsinki, Finland; Public Health England, UNITED KINGDOM

## Abstract

**Background:**

The ten-valent pneumococcal conjugate vaccine (PCV10) was introduced into the Finnish National Vaccination Program (NVP) in September 2010 using a 2+1 schedule (3, 5, 12 months). We estimated the direct and indirect effects of PCV10 on pneumonia among children to evaluate the public health impact of the vaccine.

**Methods:**

We conducted a nation-wide population-based, observational study comparing rates of pneumonia in children before and after the NVP introduction. For the total (direct and indirect) effect, the cohort of vaccine-eligible children (born June 1, 2010 or later) was followed until the end of 2013 (age range 3–42 months). For the indirect effect, a cohort of older children (age range 7–71 months) not eligible for the PCV vaccination was followed from 2011 to 2013. Both cohorts were compared with two season- and age-matched reference cohorts before NVP introduction. Hospitals’ in- and outpatient discharge notifications with ICD-10 diagnoses compatible with pneumonia (J10.0, J11.0, J12-J18, J85.1 or J86) as set by the hospital pediatricians were collected from the national Care Register. The main outcome was hospital-treated primary pneumonia (HTPP), defined as primary diagnosis of pneumonia after in-patient hospitalization. We compared rates of pneumonia in the NVP target and reference cohorts by using Poisson regression models.

**Results:**

The rate of HTPP episodes was 5.3/1000 person-years in the combined reference cohorts and 4.1/1000 person-years in the target cohort vaccine-eligible children. Compared with the reference cohort, the relative rate reduction in target cohort was 23% (95%CI 18–28) and the absolute reduction 1.3/1000 person-years. In the indirect effect evaluation, we observed continued increase in HTPP incidence until 2011 with a subsequent reduction of 18% (95%CI 10–25) during years 2012 to 2013. Number of empyema diagnoses remained low.

**Conclusions:**

A substantial decrease in pneumonia rates was observed both among vaccine-eligible children and among older, unvaccinated children after PCV10 introduction.

## Introduction

Pneumonia is the number one killer in low-income countries in children below 5 years of age [1]. In affluent countries the mortality is low, yet high disease burden remains. *Streptococcus pneumoniae* (pneumococcus) has been considered as the most common etiology in community-acquired pneumonia in children [2]. Pneumococcal conjugate vaccines have been developed to reduce this disease burden. Currently, two PCVs, 10-valent PHiD-CV10 (PCV10, GlaxoSmithKline, Belgium) and 13-valent PnCRM (PCV13, Pfizer, USA) licensed in 2009 are now being widely used throughout the world, both in affluent and low-income countries.

PCVs have been previously shown to reduce pneumonia in children. In randomized clinical trials PCV7 [3–4], PCV9 [5–6], and PCV10 [7–8] reduced both clinical and radiological pneumonia, but PCV11 only radiological pneumonia [9]. No RCTs for pneumonia have been performed in children with PCV13, which is a successor of the PCV7.

Reduction in pneumonia hospital admissions in children has been observed in many countries after large-scale introduction of the PCVs in infant vaccination programmes [10–16], although also negative results have been published [17]. There are also reports suggesting indirect impact on all-cause pneumonia for PCV7 in young adults [10] and also in longer term in older age groups as well [18], and for PCV13 [19] but to our knowledge there is no evidence of impact for PCV10 yet.

After a public tender, PCV10 was introduced into the Finnish National Vaccination Program (NVP) Program in September 2010. All children born June 1^st^, 2010 or later have been eligible for vaccination with a 2+1 schedule (vaccinations at 3, 5, and 12 months of age) without catch-up vaccinations for older age cohorts. We used the Finnish hospital discharge register data to explore the public health impact of the PCV10-NVP on pneumonia in the target group of vaccine-eligible children and also in older unvaccinated children for the indirect effect.

## Methods

We used a population-based, nation-wide observational study design as described previously [20].

**Fig 1:**
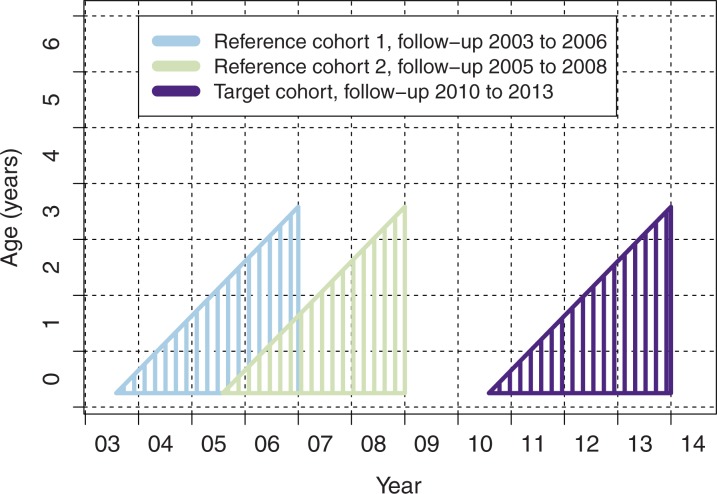
Cohorts for comparing the impact of PCV10 in vaccine-eligible children.

For total (direct and indirect) effects, we compared pneumonia rates in the vaccine-eligible cohort during the vaccination program to season- and age-matched reference cohorts before vaccine introduction (Fig 1).

**Fig 2:**
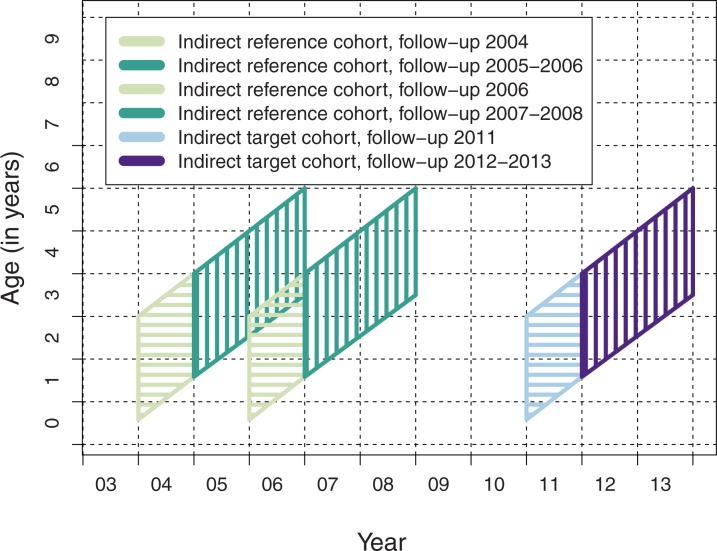
Cohorts for comparing the indirect effect of PCV10 in unvaccinated children.

For indirect effects, we compared pneumonia rates in a cohort of older children not eligible for vaccination during the vaccination program with season- and age-matched reference cohorts before vaccine introduction (Fig 2).

### Vaccination program

The NVP vaccines purchased by the government are administered at the local municipal well-baby clinics free of charge. The PCV10 vaccination coverage for children born during the calendar year 2012 was estimated in a recent survey to be 93% for the first dose and 92% for the full series of three doses [21].

Before PCV10 introduction into the NVP in 2010, use of PCVs in Finland was minimal and mostly limited to a small group of children with specific high risk conditions for whom PCV7 was recommended during 2001–2010. On the basis of national sales figures (doses distributed), the estimated uptake of PCV among children <2 years of age was less than 2% until 2009. However, a large cluster-randomized PCV10 effectiveness trial (FinIP) was conducted in Finland in 2009–2010 [22] in which more than 30,000 children under two years of age received PCV10 vaccination (approximately 22% of the corresponding birth cohort).

Influenza vaccinations were included in the Finnish NVP in fall 2007 for children 6 to 36 months of age. The annual vaccine uptake was nearly 40% until fall 2009, but considerably lower during the subsequent PCV10 era (<20%). However, during the season 2009 to 2010 the use pandemic influenza A (H1N1) vaccine uptake was considerably higher, up to 70% in children under five.

### Outcome data collection and definitions

All hospital outpatient visits and inpatient hospitalizations in Finland are registered in the *Care Register for Health Care*, a national hospital discharge register maintained by THL.

All hospitalizations and visits to hospitals associated with ICD-10 diagnoses compatible with pneumonia (J10.0, J11.0, J12 to J18, J85.1 or J86, Table 1) were identified from the Care Register. These cases were defined as hospital-diagnosed pneumonia (HDP). The main outcome was hospital-treated primary pneumonia (HTPP), i.e. a case with a primary discharge diagnosis of pneumonia after in-patient hospitalization. We also evaluated any Hospital-diagnosed pneumococcal pneumonia defined as any register-based event with an ICD10 diagnosis compatible with pneumococcal pneumonia (ICD-10 code J13); and Empyema defined as ICD10 code J86 and hospitalization at least overnight.

**Table 1:** ICD-10 codes compatible with pneumonia and their distribution in the two reference cohorts from 2003 to 2006 and 2005 to 2008.

ICD-10 code	Diagnosis in text	Proportion of episodes in the reference cohorts, %
**J10.0**	Influenza with pneumonia, virus identified	1.1
**J11.0**	Influenza with pneumonia, virus not identified	0.4
**J12**	Viral pneumonia	7.9
**J13**	Pneumonia due to *Streptococcus pneumoniae*	2.5
**J14**	Pneumonia due to *Haemophilus influenzae*	0
**J15**	Bacterial pneumonia, not elsewhere classified	20.0
**J16**	Pneumonia due to other infectious organisms	0.1
**J17**	Pneumonia in diseases classified elsewhere	0.1
**J18**	Pneumonia, organism unspecified	67.5
**J85.1**	Abscess of lung with pneumonia	0.1
**J86**	Pyothorax, including empyema	0.3

No radiological data were available for evaluation of the pneumonia cases. No results of any diagnostic tests performed in the hospital were available. However, pneumonia cases were linked with the blood culture confirmed cases reported to the National Infectious Diseases Register to detect cases of bacteremic pneumonia.

We used a 90-day period starting from the first pneumonia event to combine potential multiple events into one episode.

### Cohorts and follow-up for assessment of total (direct and indirect) effects

To compare pneumonia rates before and after NVP introduction we constructed PCV10 target and reference cohorts by using the data from the Population Information System as previously reported [20].

Vaccine-eligible children were defined as those with birthdates from June 2010 to September 2013 (PCV10 target cohort, Fig 1) irrespective of whether they had received the vaccine or not. For the assessment of total effects of PCV10, two age- and season-matched reference cohorts were formed of children born June 2003—September 2006 (Reference cohort 1, Fig 1), and June 2005—September 2008 (Reference cohort 2, Fig 1). In each of the cohorts, follow-up started at three months of age (i.e., the expected age at the first dose) and ended between the ages of 3 to 42 months.

To minimize the influence of the FinIP trial vaccinations [22] in the reference cohorts, the cohorts were formed in such a way that the years 2009–2010 were not included in the follow up (Fig 1).

### Cohorts and follow-up for assessment of indirect effects

For the evaluation of indirect PCV10 effects, the unvaccinated cohort of children included those born from January 2008 through May 2010 (target cohort for indirect effects, Fig 2). Children vaccinated with PCV10 in the cluster-randomized PCV10 effectiveness trial [22] were excluded. For the comparison, two age and season matched reference cohorts were formed that included children born from January 2001 through May 2003, and from January 2003 through May 2005 (Fig 2) with follow-up between 7 to 71 months of age.

For the analysis, the follow-up time was divided into two periods to assess the development of the indirect effect after PCV10 introduction. In the target cohort, the first period included year 2011 and the second period years 2012 to 2013 (Fig 2). These time periods were compared with the age- and season-matched time periods in the two reference cohorts (Fig 2).

Year 2014 data were included in the descriptive analyses for both vaccinated and unvaccinated populations.

### Statistical analysis

Pneumonia rates in the target cohort were compared to rates in the combined reference cohorts using Poisson regression models. Numbers of pneumonia cases in the cohorts during the observation periods were tabulated and the corresponding person-years of follow-up were used as weights in the analysis. Relative rate reduction (percent) was calculated as (1 –relative risk)*100%. In case of zero cases in either cohorts, ratio of Poisson means was derived conditional on total number of cases [23] and Clopper-Pearson confidence interval for the resulting binomial distribution was used [24]. Absolute rate reductions and the corresponding 95% confidence intervals were calculated from the model estimates using the delta method.

The study protocol was approved by the THL institutional review board. Permission to use the register data were obtained from the relevant register controllers at THL.

## Results

**Fig 3:**
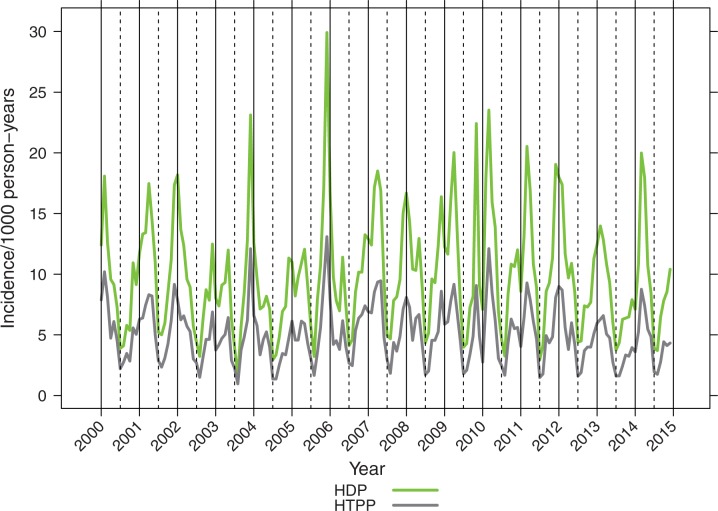
Monthly incidence rates of pneumonia in children <24 months of age from 2000 through end 2014 with vertical lines showing calendar years and dashed vertical lines showing epidemiological years.

**Fig 4:**
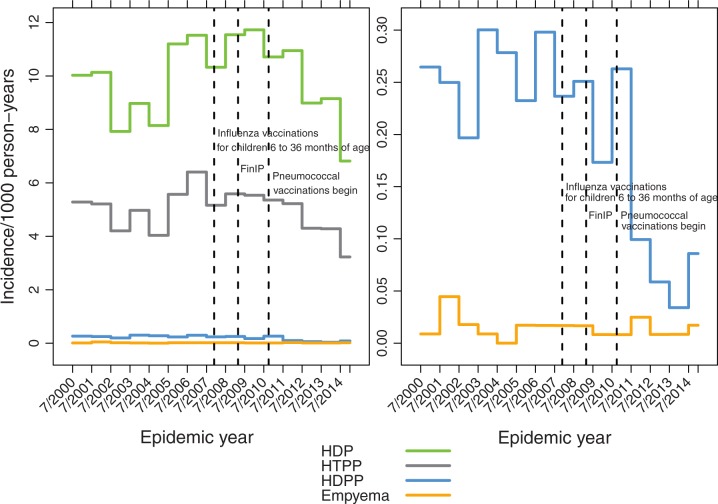
Annual pneumonia rates in children less than 2 years of age by epidemic years. A) all pneumonia outcomes, HDP hospital-diagnosed pneumonia, HTPP hospital-treated primary pneumonia, HDPP hospital-diagnosed pneumococcal pneumonia and empyema; B) HDPP and empyema on different scale.

**Fig 5:**
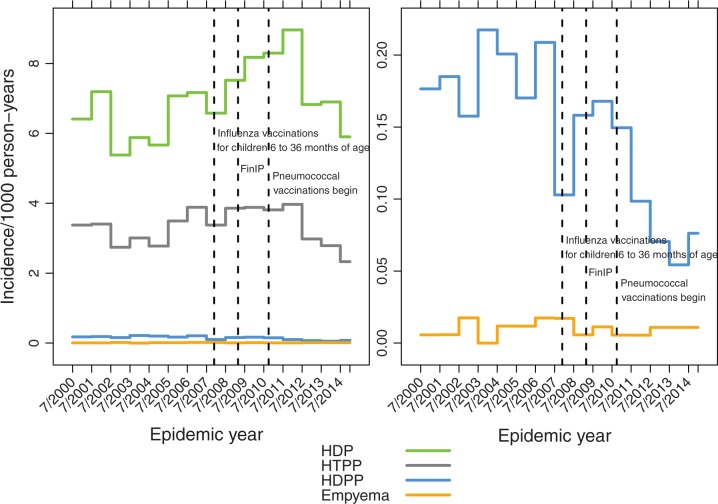
Annual pneumonia rates in children 2 to 4 years of age by epidemic years. A) all pneumonia outcomes, HDP hospital-diagnosed pneumonia, HTPP hospital-treated primary pneumonia, HDPP hospital-diagnosed pneumococcal pneumonia and empyema; B) HDPP and empyema on different scale.

The incidence of pneumonia showed marked seasonal fluctuation, peaks occurring in late fall, winter, or early spring months from October through March, varying in magnitude and timing each year (Fig 3). To reduce the effect of this variation on annual estimates yearly rates were calculated by epidemic years (July to June). From 2003/2004 through 2009/2010 the incidence of hospital-diagnosed pneumonia fluctuated considerably and increased in children <5 years of age (Figs 4 and 5). After the PCV introduction, pneumonia rates decreased substantially in 2012 to 2014 (Figs 4 and 5), but empyema rates remained stable.

### Total (direct and indirect) impact of PCV10 in children

The analysis included 334 087 child-years of follow-up in the target cohort eligible for vaccination; the rate of any pneumonia (HDP) episodes was 9.0/1000 person-years compared with 10.3/1000 person-years in the combined reference cohorts (relative rate reduction 13% (95%CI 9–16); absolute rate reduction 1.3/1000 person-years, Table 2).

**Table 2:** Rates of pneumonia and the corresponding rate reductions in PCV10 eligible target cohort vs. reference cohorts.

	Incidence rate /1000 in reference cohorts (No. of cases)		Incidence rate /1000 in target cohort (No. of cases)	Relative rate reduction (95% CI)	Absolute rate reduction (95% CI)
	2003–06^1^	2005–08^1^	2010–2013^2^	Target vs. reference cohorts combined	Target vs. reference cohorts combined
Hospital-diagnosed pneumonia	9.8 (3141)	10.8 (3549)	9.0 (3004)	13 (9, 16)	1.3 (0.9, 1.7)
Hospital-treated primary pneumonia	5.1 (1626)	5.6 (1838)	4.1 (1364)	23 (18, 28)	1.3 (1.0, 1.5)
Hospital-diagnosed pneumococcal pneumonia	0.23 (73)	0.27 (88)	0.06 (19)	77 (64, 86)	0.2 (0.1, 0.2)
Empyema	0.016 (5)	0.015 (5)	0.015 (5)	3 (-174, 70)	0.0 (-0.02, 0.02)
Vaccine-type bacteremic pneumonia	0.12 (38)	0.12 (39)	0.015 (5)	87 (72, 96)	0.1 (0.07, 0.13)

1) Follow-up years in the two reference cohorts 649,877, age 3–42 months, born Jun’03-Sep’06 or Jun’05-Sep’08.

2) Follow-up years in the target cohort 334,087, age 3–42 months, born Jun’10-Sep’13.

**Fig 6:**
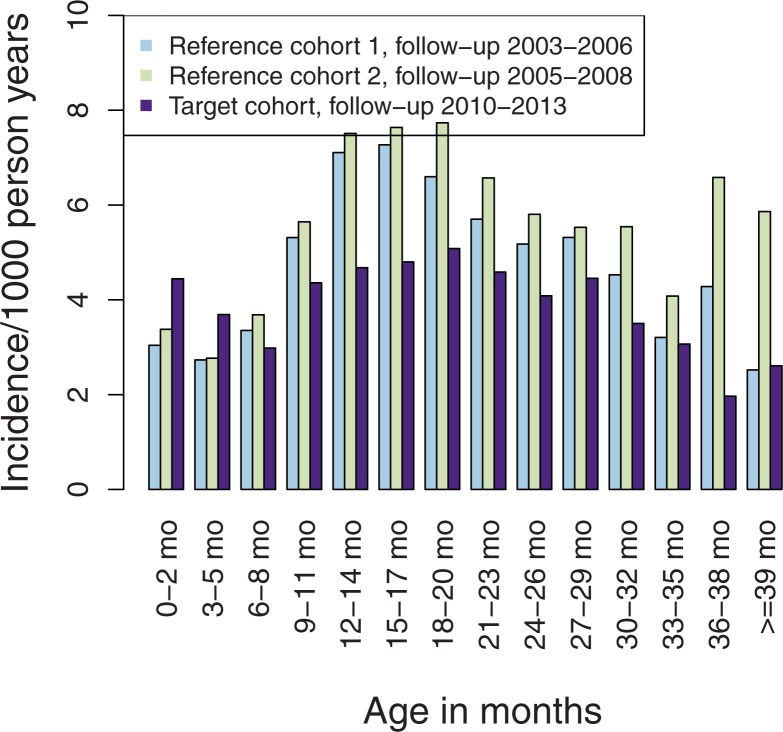
The incidence of hospital-treated primary pneumonia (HTPP) by age in vaccine-eligible target cohort compared to reference cohorts before introduction.

For HTPP, the incidences were roughly half of HDP with the relative rate reduction of 23% (95%CI 18–28) in the vaccine-eligible cohort compared to the reference cohorts and a similar absolute rate reduction as for any HDP. The incidence of HTPP by age is shown in Fig 6. The incidence was lower in the target cohort in all age groups from 6–8 months of age (i.e. after two doses) through 39–42 months of age.

There were 5 cases of empyema in both of the two reference cohorts (0.2% of all pneumonia cases). After NVP introduction, similar incidence with 5 cases was observed also in the target cohort eligible for vaccination; relative rate reduction was 3% (95%CI -174 to 70).

Vaccine-type bacteremic pneumonia was recorded only in 5 children in the target cohort. The relative rate reduction was 87% (95% CI 72 to 96, Table 2).

### Indirect PCV10 effects in unvaccinated children

The pneumonia rates/1,000 person-years for the follow-up year 2011 and 2012/2013 combined are presented in Table 3.

**Table 3:** Rates of pneumonia and the corresponding rate reductions in unvaccinated study cohort vs. reference cohorts.

		Incidence rate/1000 in reference cohorts (No. of cases)		Incidence rate /1000 in target cohort (No. of cases)		Relative rate reduction for the indirect effect (95% CI)		Absolute rate reduction for the indirect effect (95% CI)	
	Post introduction year	2004&2006^1^	2005–06& 2007–08^2^	2011^3^	2012–2013^4^	2011 vs. 2004&2006	2012–13 vs. 2005–06 & 2007–08	2011 vs. 2004&2006	2012–13 vs. 2005–06 & 2007–08
**Hospital-diagnosed pneumonia**	Year one	8.1 (1007+1242)		11.9 (1452)		-46 (-56, -36)		-3.7 (-4.4, -3.0)	
	Years 2–3		6.3 (1600+1878)		6.3 (1561)		-1 (-7, 5)		-0.1 (-0.4, 0.3)
**Hospital-treated primary pneumonia**	Year one	4.4 (536+669)		5.5 (669)		-25 (-38, -14)		-1.1 (-1.6, -0.6)	
	Years 2–3		3.2 (811+972)		2.6 (653)		18 (10, 25)		0.6 (0.3, 0.8)
**Hospital-diagnosed pneumococcal pneumonia**	Year one	0.27 (49+25)		0.25 (30)		9 (-38, 41)		0.02 (-0.08, 0.13)	
	Years 2–3		0.18 (56+42)		0.05 (13)		70 (49, 84)		0.12 (0.08, 0.17)
**Empyema**	Year one	0.02 (2+3)		0.01 (1)		55 (-180, 98)		0.01 (-0.01, 0.03)	
	Years 2–3		0.01 (2+2)		0 (0)		100 (-240, 100)		0.01 (0, 0.01)

^1^ Follow-up 136 174+139 820 person-years, age 7–48 months, born Jan’01-May’03.

^2^Follow-up 273 352+281 012 person-years, age 19–71 months, born Jan’01-May’03 or Jan’03-May’05.

^3^ Follow-up 122 331 person-years, age 7–48 months, born Jan’08-May’10.

^4^ Follow-up 246 773 person-years, age 19–71 months, born Jan’08-May’10.

There was no reduction observed in calendar year 2011 in unvaccinated children 7–48 months of age. However, in 2012 and 2013 combined, there was a statistically significant reduction of 18% in hospital-treated primary pneumonia and 70% in pneumococcal pneumonia in unvaccinated children 19–71 months of age. This reduction was especially prominent in children below 4 years of age with reversal of the increasing trend (S1 Fig).

## Discussion

We observed an increasing trend in register-based pneumonia episodes before the PCV10 introduction into the Finnish National Vaccination Programme followed by a significant decrease after it. Similar trends were seen for both the vaccinated and unvaccinated populations. No increase in empyema rates was detected in the follow-up until end 2014; however, the number of cases was low.

In clinical trials with PCV7, PCV9, PCV10, and PCV11 the VE estimates in the reduction of clinical pneumonia have ranged from -1 to 17% [3–7, 9]. However, the study population, outcome detection methods, case definitions and vaccination schedules differ from trial to trial. A higher point estimate of 27% was observed in the FinIP trial conducted in Finland just prior to the introduction of PCV into the NVP [8]. Furthermore, identical case definitions and detection methods were used in the FinIP trial and the current study. The results are concordant, yet the reduction in hospital-diagnosed pneumonia was lower in the current study compared to the trial. This may be due to increasing trend in pneumonia diagnoses observed prior to NVP implementation.

Our estimates are compatible with observational studies from Brazil [12, 25–26] and Chile [16] after introduction of PCV10 as the first PCV, although in these countries the vaccine was introduced with a 3+1 schedule. However, also much higher reductions in clinical pneumonia have been reported in before-after studies ranging from 13% to up to 78% [10–11, 27–29]. Although higher reductions in national vaccination programmes with high vaccination coverage can be obtained than in clinical trials (due to the development of additional indirect impact), it is difficult to foresee, how most of the clinical pneumonia could be prevented with the current PCVs. The maximum expected reduction in overall clinical pneumonia might be 32% (assuming 50% of pneumonia caused by pneumococcus, 85% vaccine-type (+vaccine-related type) coverage based on invasive pneumococcal disease data [20] since no data on pneumonia are available, VE 85% against vaccine-type pneumonia, and modest 50% increase in non-vaccine-type pneumococcal pneumonia, i.e. replacement disease, see S1 Table). Any observation above that would most likely be biased due to other factors than vaccine impact. Naturally, more specific outcomes for pneumococcal disease like consolidated pneumonia would result in higher relative reductions and more sensitive outcomes like any clinical pneumonia including primary care diagnoses would result in lower estimates.

Looking at S1 Table in another way, the current relative and absolute reduction estimates for HDP would be expected with following assumptions using invasive pneumococcal disease as a reference [20](28% of pneumonia caused by pneumococcus, 81% vaccine-type coverage, VE 70% against vaccine-type pneumonia, and 85% increase in non-vaccine-type pneumonia). Different scenarios and examples from the current study are shown in S1 Table.

In addition to the well-known seasonal fluctuation in pneumonia due to viral epidemics, there were also considerable year-to-year fluctuations of the pneumonia incidences. Upon exploration of the National Infectious Diseases Register data [30] for various respiratory tract pathogens during these calendar years for children under 5 years of age, influenza showed highest peaks in 2003 and 2009, respiratory syncytial virus (RSV) in 2005, 2010, and 2014, Mycoplasma pneumoniae in 2011 to 2012, and Bordetella pertussis in 2003 to 2004. These were not closely associated with peaks in pneumonia in calendar years 2001, 2005, and 2007. The absence of influenza in calendar years 2004 and 2010 was associated with a low pneumonia incidence only in 2004. The yearly fluctuation was lower when epidemic years, rather than calendar years were evaluated (Fig 4, S2 Fig).

Our study was a nation-wide population-based study, including all hospitals treating pediatric patients in Finland. Through register-linkage and the use of personal identity code, which is assigned to each resident in Finland and is unique and permanent, allowed close to complete data collection.

However, as any observational study, also ours is prone to bias due to secular trends and changes in diagnostic, coding, and treatment practices. Two reference cohorts were selected to reduce the effect of short-term secular trends and enabled calculation of robust baseline rates. Pediatric hospital outpatient visits have increased from years 2003–2007 to years 2010–2013 by 10.5% (from 380 to 420 visits per 1000 children under 15 years of age, www.sotkanet.fi). On the other hand, the corresponding inpatient admissions have decreased from 106 admissions to 96 admissions per 1000 children by 2013. Thus, the net change for all hospital visits and admissions combined have increased from 486 to 516, i.e. increase of 6.2%. The increasing trend prior to the vaccine introduction was also evident for any hospital-diagnosed pneumonia. Since we did not use any adjustment for this trend, our estimates are conservative.

We compared the vaccine-eligible population with a high vaccination coverage (93%), but not 100%, and some subjects (estimated below 2%) in the unvaccinated reference population had actually received pneumococcal vaccine. Thus, our estimate for the total impact is an underestimate of the true vaccine effect. On the other hand, the target cohort for the indirect impact assessment had probably more commonly received a pneumococcal vaccine (estimated less than 2% for birth cohort 2008 and less than 10% for birth cohort 2009 and 1-5/2010) than the corresponding reference cohorts. However, as there was no impact seen in 2011, but only in 2012 to 2013, it is more probably due to the NVP indirect impact than direct impact of the vaccinations in older children.

We were not able to collect data on the etiology on the pneumonia episodes. First, those data are not in the registers (except for bacteremic cases), and secondly, routine microbiological diagnostics, like blood cultures, lack sensitivity and they do not very often yield a specific etiology. Finally, the microbiological assays also suffer from poor specificity, like urine antigen assays [31], or from poor feasibility, like lung taps. Therefore, nearly all pneumonia cases are treated empirically, which is also reflected in the non-specific pneumonia codes found in the data (Table 1). We included all ICD-10 codes for pneumonia, also those for viral pneumonia, to reduce any potential bias due to changes in coding practices within the pneumonia diagnoses.

Furthermore, we relied upon register-based diagnoses and no formal radiological evaluation was performed. However, based on FinIP trial data, roughly 90% of pediatric pneumonia patients in Finland have a chest X-ray available [8] taken on clinical indications; two thirds of the HTPP episodes were confirmed positive in retrospective radiological evaluation.

Finally, although we collected hospital outpatient pneumonia visits, we did not have data on pneumonia diagnoses at the public or private ambulatory care outside hospitals. Therefore, our data represents more severe cases of pneumonia requiring hospital referral.

To our knowledge, this is the first nation-wide population-based impact study to document the direct and indirect effects of routine PCV10 vaccination among vaccine-eligible and unvaccinated children. Surveillance data in the coming years will shed more light on the full potential of PCV10 in reducing pneumonia and other pneumococcal disease in the unvaccinated population.

## Supporting information

S1 Table Projected impact against pneumonia in different scenarios and examples from the current study with defined absolute and relative reductions for pneumonia to estimate proportion of pneumococcal pneumonia and vaccine effectiveness estimates. (DOC)

S1 Fig The incidence of hospital-treated primary pneumonia (HTPP) by age in cohorts of unvaccinated children for evaluation of the indirect impact. (PNG)

S2 Fig Annual pneumonia rates in children less than 2 years of age by calendar years. A) all pneumonia outcomes, HDP hospital-diagnosed pneumonia, HTPP hospital-treated primary pneumonia, HDPP hospital-diagnosed pneumococcal pneumonia and empyema;B) HDPP and empyema on different scale.
(DOC)
