# Supplementary material for: Impact of ten-valent pneumococcal conjugate vaccine on pneumonia in Finnish children in a nation-wide population-based study
Source: PLoS One. 2017 Mar 1;12(3):e0172690. doi: 10.1371/journal.pone.0172690 (PMC5332024; doi:10.1371/journal.pone.0172690)
Supplement: S1 Table — (DOC) [file pone.0172690.s001.doc]

**S1 Table. Projected impact against pneumonia in different scenarios and examples from the current study with defined absolute and relative reductions for pneumonia to estimate proportion of pneumococcal pneumonia and vaccine effectiveness estimates**

|  | **Before vaccination** | | | | | **Vaccine impact estimates** | | | **After vaccination** | | | |
| --- | --- | --- | --- | --- | --- | --- | --- | --- | --- | --- | --- | --- |
|  | A | B | C | D | E | F | G | H | I | J | K | L |
| Variable | Incidence rate | Proportion of pnc pneumonia | Incidence of pneumococcal pneumonia | Vaccine serotype coverage | vaccine-related serotype coverage | VE estimate, VT disease | VE estimate, VR disease | Increase in non-VT disease | Reduction in VT and VR disease | Increase due to non-VT disease | Absolute reduction in pneumonia | Relative reduction in overall pneumonia |
| unit | per 1000 person-years | fraction | per 1000 person-years | fraction | fraction | fraction | fraction | fraction | per 1000 person-years | per 1000 person-years | per 1000 person-years | percentage |
| **Scenario** | estimated | | A*B | estimated | | estimated | | | C*(D*F+E*G) | C*(1-D-E)*H | I-J | K/A*100 |
| Maximal impact^1^ | 10.0 | 0.5 | 5.0 | 0.80 | 0.05 | 0.85 | 0.85 | 0.5 | 3.61 | 0.38 | 3.23 | 32 |
| **Median impact** | **10.0** | **0.35** | **3.5** | **0.70** | **0.05** | **0.7** | **0.7** | **0.75** | **1.84** | **0.66** | **1.18** | **12** |
| Minimal impact^2^ | 10.0 | 0.25 | 2.5 | 0.60 | 0.05 | 0.6 | 0.5 | 1 | 0.96 | 0.88 | 0.08 | 1 |
| **After full eradication of VT and VR disease** | **10.0** | **0.35** | **3.5** | **0.70** | **0.05** | **1** | **1** | **1** | **2.63** | **0.88** | **1.75** | **18** |
| **Examples from the current study** | reported | fitted into data | A*B | reported for IPD^3^ | | fitted into data | | reported for IPD^3^ | C*(D*F+E*G) | C*(1-D-E)*H | reported | reported |
| HDP | 10.29 | 0.28 | 2.9 | 0.81 | 0.04 | 0.7 | 0.5 | 0.85 | 1.69 | 0.37 | 1.3 | 13 |
| HTPP | 5.33 | 0.51 | 2.7 | 0.81 | 0.04 | 0.7 | 0.5 | 0.85 | 1.60 | 0.35 | 1.3 | 23 |

pnc, pneumococcal; VE, Vaccine Efficacy/Effectiveness; VT, Vaccine Type; VR, vaccine-related serotype;

NOTES

Replament due to other etiologies than pneumococcus not estimated, calculations based on non-biased point estimates

^1^ Maximal impact assumes high proportion of pneumonia due to pneumococcal etiology, high vaccine type proportion within pneumococcal pneumonia and high vaccine effectiveness against pneumonia

^2^ Minimal impact assumes low proportion of pneumonia due to pneumococcal etiology, low vaccine type proportion within pneumococcal pneumonia and low vaccine effectiveness against pneumonia

^3^ Reference 20: Jokinen J, Rinta-Kokko H, Siira L, Palmu AA, Virtanen MJ, Nohynek H, et al. Impact of ten-valent pneumococcal conjugate vaccination on invasive pneumococcal disease in Finnish children-a population-based study. PLoS One. 2015;10:e0120290. doi: 10.1371/journal.pone.0120290. eCollection 2015.
