# Supplementary figures and images for: Impact of ten-valent pneumococcal conjugate vaccine on pneumonia in Finnish children in a nation-wide population-based study
Source: PLoS One. 2017 Mar 1;12(3):e0172690. doi: 10.1371/journal.pone.0172690 (PMC5332024; doi:10.1371/journal.pone.0172690)

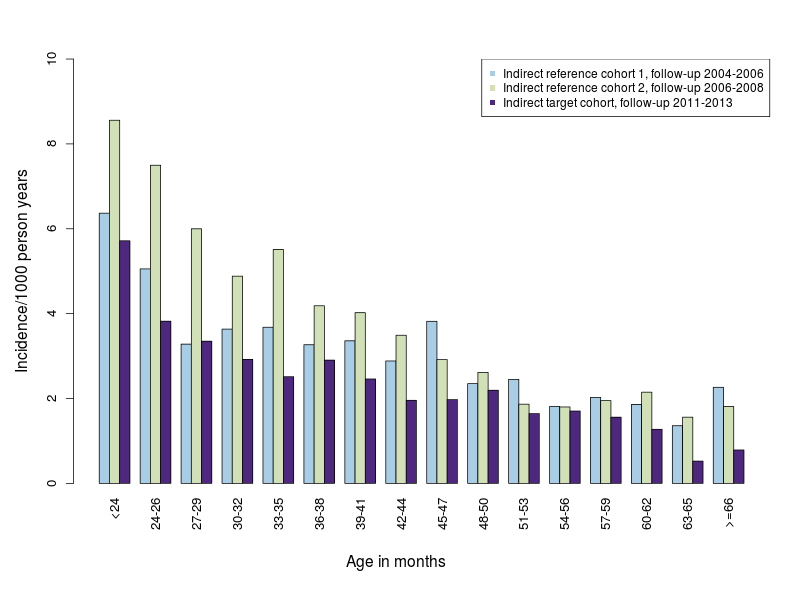

Supplement: S1 Fig — (PNG) [file pone.0172690.s002.png]
