# Supplementary material for: Impact of ten-valent pneumococcal conjugate vaccine on pneumonia in Finnish children in a nation-wide population-based study
Source: PLoS One. 2017 Mar 1;12(3):e0172690. doi: 10.1371/journal.pone.0172690 (PMC5332024; doi:10.1371/journal.pone.0172690)

**S2 Figure: Annual pneumonia rates in children less than 2 years of age by calendar years**

1. **all pneumonia outcomes, HDP hospital-diagnosed pneumonia, HTPP hospital-treated primary pneumonia, HDPP hospital-diagnosed pneumococcal pneumonia and empyema;**
2. **HDPP and empyema on different scale**

A


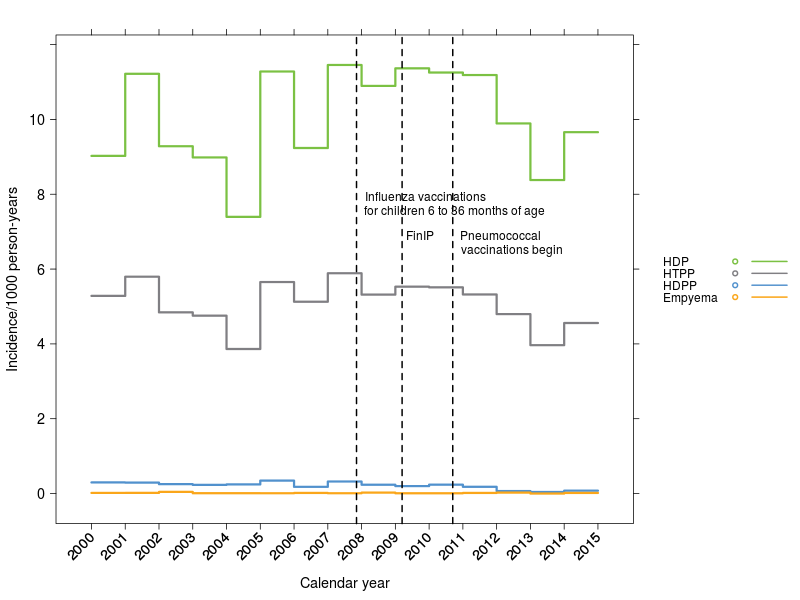


B


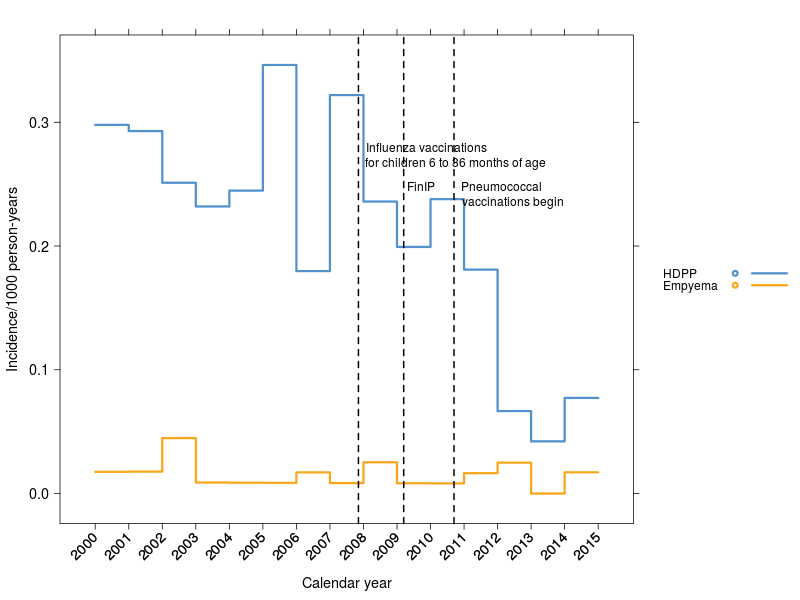

Supplement: S2 Fig — A) all pneumonia outcomes, HDP hospital-diagnosed pneumonia, HTPP hospital-treated primary pneumonia, HDPP hospital-diagnosed pneumococcal pneumonia and empyema;B) HDPP and empyema on different scale. (DOC) [file pone.0172690.s003.doc]
